# Prognostic Significance of RAN Expression and RAN-Associated Pathways in Breast Cancer: A Systematic Review and Exploratory Meta-Analysis

**DOI:** 10.3390/cancers18213591

**Published:** 2026-11-09

**Authors:** Mohamed El-Tanani, Syed Arman Rabbani, Mohamedanas Mohamedfaruk Patni, Rasha Aziz Attia Salama, Adil Farooq Wali, Riham A. Ibrahim, Yahia El-Tanani, Mohammad Isreb

**Affiliations:** 1 RAK College of Pharmacy, RAK Medical and Health Sciences University, Ras Al Khaimah 11172, United Arab Emirates; 2 RAK College of Medical Sciences, RAK Medical and Health Sciences University, Ras Al Khaimah 11172, United Arab Emiratesriham@rakmhsu.ac.ae (R.A.I.); 3 Royal Cornwall Hospitals, NHS Trust, Treliske, Truro TR1 3LJ, UK; 4 School of Pharmacy and Medical Sciences, University of Bradford, Bradford BD7 1DP, UK

**Keywords:** RAN expression, Ran GTPase, breast cancer, prognosis, biomarker, patient stratification, survival, metastasis, systematic review, meta-analysis

## Abstract

**Background**: RAN is a small GTPase that regulates nucleocytoplasmic transport, mitotic spindle assembly and chromosome segregation. Experimental studies implicate increased RAN pathway activity in breast cancer proliferation, invasion, metastasis and treatment resistance, whereas clinical studies have primarily measured tumour RAN protein or mRNA expression. The independence and quantitative consistency of this clinical evidence have not been systematically assessed. **Methods**: PubMed, Embase, Scopus and Web of Science were searched from inception to 1 September 2026 using separate direct RAN and pathway extension strategies. Two reviewers independently screened titles/abstracts and full texts, with disagreements resolved by consensus. One reviewer extracted data using a piloted template and a second reviewer checked every entry; discrepancies were resolved by consensus. Cohort overlap was assessed at institutional cohort and GEO accession levels. Published survival effect estimates were extracted for direct protein expression studies, and unique GEO datasets were reanalysed using a prespecified within-cohort median RAN cut-off and univariable Cox regression. Endpoint-specific random-effects models used restricted maximum likelihood with Hartung–Knapp confidence intervals. Direct evidence measured RAN protein or mRNA; canonical axis evidence assessed established RAN-dependent components or predefined signatures; peripheral/historical links based on nomenclature, paralogy or earlier interaction evidence were contextual only. QUIPS and QUADAS-2 were applied separately, and certainty was assessed using GRADE guidance adapted for prognostic factor research. **Results**: The eight database exports contained 891 records. After removal of 352 duplicates, 539 unique records were screened; 23 full-text reports were assessed, and 15 clinical publications were included. Six publications were classified as direct RAN, seven as canonical RAN-axis evidence and two as peripheral/historical RAN-associated evidence. The primary protein evidence comprised one Liverpool estimate (Liverpool cohort n = 288; reported Cox RR 12.53, 95% CI 3.95–39.76) and Sheng et al. (multivariable n = 123; HR 2.72, 95% CI 1.15–6.42); these were displayed separately. Across three unique GEO cohorts per endpoint, pooled HRs for high versus low RAN mRNA were 1.38 (95% CI 0.78–2.45; tau-squared = 0) for distant metastasis-free survival, 1.20 (0.36–4.04; tau-squared = 0.1222) for recurrence-free survival, and 1.35 (0.69–2.67; tau-squared = 0) for overall survival. Certainty was low for protein, distant metastasis-free survival, and overall survival, and very low for recurrence-free survival and subgroup findings. Canonical target-specific evidence was not pooled. **Conclusions**: Adverse protein–survival associations rest on only two independent retrospective cohorts and do not establish a stable effect magnitude or clinical utility; the pooled mRNA findings were inconclusive and the subgroup findings were exploratory. Overlapping cohorts, heterogeneous assays and cut-offs, and the small number of independent datasets limit certainty. The findings support further biomarker development, but prospective multicentre validation and assay standardisation are required before any clinical application.

## Simple Summary

Breast cancers with similar routine test results can have different outcomes, creating a need for better markers of recurrence and survival. We reviewed 15 clinical publications on a protein called RAN and related cellular pathways, carefully checking whether different papers reused the same patients. Two independent studies of tumour protein levels linked higher RAN to poorer survival. However, combined analyses of public datasets measuring gene activity did not provide conclusive results. The evidence was limited by small numbers of independent patient groups, differing laboratory methods and mostly retrospective studies. RAN may deserve further study alongside established markers and in carefully designed combinations with other proteins. It is not ready to guide treatment. Independent studies using standardised tests must establish whether RAN adds useful information beyond current clinical and molecular assessments.

## 1. Introduction

Breast cancer is the most frequently diagnosed malignancy in women and remains a major cause of cancer mortality worldwide [1]. Its clinical course is heterogeneous even within groups defined by tumour size, nodal status, histological grade, oestrogen receptor (ER), progesterone receptor (PR), human epidermal growth factor receptor 2 (HER2) and proliferation markers. Molecular classification has improved biological understanding and treatment selection, but recurrence and metastatic progression remain difficult to predict for individual patients [2,3]. Additional biomarkers are therefore needed to refine prognosis, identify biologically high-risk disease within conventional clinical groups and support the development of biomarker-directed treatment strategies.

RAN (Ras-related nuclear protein) is a highly conserved small GTPase. A spatial RAN-GTP/RAN-GDP gradient, established principally by the chromatin-associated guanine nucleotide exchange factor RCC1 and cytoplasmic RAN-GTPase-activating proteins, coordinates nuclear transport, spindle assembly and chromosome segregation [4]. These functions are especially relevant to rapidly dividing malignant cells. RAN signalling also intersects with oncogenic programmes that regulate survival, migration and invasion, including PI3K/AKT/mTOR, RAS/MEK/ERK, MYC, and MET-associated pathways [5,6,7,8].

Clinical breast cancer studies have evaluated RAN at several biological levels. Immunohistochemical studies measured nuclear and cytoplasmic RAN protein, whereas transcriptomic studies assessed RAN mRNA or RAN pathway components. Other reports examined whether RAN becomes more prognostically informative when interpreted with MYC, MET, matrix metalloproteinase 2 (MMP2), nodal status, receptor status or triple-negative phenotype [6,7,8,9,10,11,12]. These approaches address related but distinct questions. Direct RAN expression concerns the prognostic value of the biomarker itself, while paired-marker and pathway studies explore biological context and patient stratification.

A major methodological problem is that the published literature is not composed of independent patient cohorts. Several publications reuse the same or related Liverpool tissue series, and multiple transcriptomic reports analyse overlapping GEO datasets. Treating each publication as an independent study would double-count patients and produce artificially precise pooled estimates. The present review therefore used the cohort or public dataset, rather than the publication, as the statistical unit.

This systematic review aimed to evaluate the prognostic significance of tumour RAN expression and the clinical evidence for selected RAN-associated pathway markers in breast cancer. The primary objective was to assess whether elevated tumour RAN expression is associated with poorer survival. Secondary objectives were to examine cellular localisation, molecular and clinicopathological strata, RAN-associated targets, and the extent to which the available evidence supports further clinical biomarker development.

## 2. Materials and Methods

### 2.1. Review Design and Reporting

The review was designed as a prognostic factor systematic review with an exploratory meta-analysis. Supporting Information is organised as Supplementary File S1 (search and study selection records), Supplementary File S2 (quantitative synthesis details), Supplementary File S3 (risk-of-bias and certainty assessments), Supplementary File S4 (data-extraction template), Supplementary File S5 (GEO reanalysis and effect-measure audits), and Supplementary File S6 (pathway evidence mapping). The reporting of this systematic review and meta-analysis was guided by the Preferred Reporting Items for Systematic Reviews and Meta-Analyses (PRISMA 2020) statement, the PRISMA extension for reporting literature searches (PRISMA-S), and the Meta-analysis of Observational Studies in Epidemiology (MOOSE) guidelines [13,14,15]. Prognostic and clinicopathological studies were assessed across the six QUIPS domains: study participation, attrition, prognostic factor measurement, outcome measurement, confounding, and statistical analysis/reporting [16]. For the clinicopathological reports by Behrens, Emberley and Siddig, the six QUIPS domains were operationalised for the study design: participation assessed specimen selection; attrition assessed specimen, assay and clinical annotation completeness; factor measurement assessed marker assay/scoring; outcome measurement assessed pathology or receptor classification; confounding assessed differences, such as age, grade and subtype; and analysis/reporting assessed comparison methods, multiplicity and precision. Longitudinal attrition was not applicable, and absence of follow-up was not itself taken as evidence of low bias. This descriptive adaptation is not a separately validated instrument and supports narrative clinicopathological synthesis only. Domain criteria and study-specific rationales are supplied in Supplementary File S3. The RANBP3 methylation diagnostic study was assessed separately with QUADAS-2 because its principal outcome was diagnostic discrimination [17]. QUADAS-2 risk-of-bias judgements (four domains) and applicability concerns (three domains) were kept separate from QUIPS; insufficient reporting was labelled unclear, rather than moderate. Overall QUIPS judgements were classified as low when all domains were low, moderate when at least one domain was moderate and none was high, and high when at least one domain was high. No numeric quality score was calculated. REMARK principles guided biomarker-reporting appraisal [18]. Certainty for each main outcome was assessed using GRADE guidance adapted for prognostic factor research, considering phase of investigation, risk of bias, inconsistency, indirectness, imprecision and publication bias [19,20].

The review question was framed using PECO: patients with breast cancer constituted the population; high tumour RAN expression was the exposure; low tumour RAN expression was the comparator; and survival and clinicopathological outcomes were the outcomes of interest. Analyses of RAN-associated markers were treated as exploratory contextual evidence rather than as interchangeable measures of RAN. The review protocol was prospectively registered on the Open Science Framework (https://doi.org/10.17605/OSF.IO/BV3NG).

### 2.2. Search Strategy

PubMed, Embase, Scopus, and Web of Science Core Collection were searched from inception to 1 September 2026. Two complementary strategies were run in each database: a direct RAN search and a pathway extension search. The direct search combined RAN identifiers with breast cancer terms without a mandatory outcome filter, because prognostic information was often reported only in full text. The extension search combined predefined RAN-associated nuclear transport markers (RANBP1, RANBP3, RANBP3L, RANBP9/RanBPM, CSE1L/CAS, XPOT, and XPO5) with breast cancer terms. No date, language or study design filters were applied. Complete database-specific strings, search dates and yields are supplied in Supplementary File S1. Reference lists and citation links from eligible studies and relevant reviews were also checked. The PRISMA checklist can be found in the Supplementary Materials.

### 2.3. Eligibility Criteria

Studies were eligible when they reported original human breast cancer data and patient-level survival, diagnostic or clinicopathological stratification outcomes. For synthesis, evidence was classified into three analytical tiers: (1) direct RAN studies measuring tumour RAN protein or RAN mRNA; (2) canonical RAN-axis studies evaluating an established RAN-dependent transport or regulatory component, or a predefined RAN pathway signature, in clinical specimens or annotated patient datasets; and (3) peripheral or historical RAN-associated studies evaluating proteins linked to RAN by nomenclature, paralogy or earlier interaction evidence but not interchangeable with canonical RAN-axis markers. Tier 3 studies were retained only for contextual narrative synthesis and were excluded from pooled estimates and core clinical conclusions. Markers in all tiers were required to have clinical stratification data and were never treated as interchangeable measures of RAN. Retrospective tissue cohorts and secondary analyses of public transcriptomic cohorts were eligible. Cell line-only, animal-only and in silico-only studies without an eligible patient outcome were excluded, although mechanistic components of mixed clinical-translational studies were used for interpretation. Reviews, editorials, conference abstracts without sufficient full data, studies of non-breast malignancies, and germline susceptibility studies comparing cases with cancer-free controls were excluded.

Two reviewers independently screened all titles and abstracts and then independently assessed potentially eligible full texts. Disagreements at either stage were resolved by consensus. Reasons for exclusion were recorded at the full-text stage.

### 2.4. Outcomes and Effect Measures

The primary outcome was overall or patient survival. Secondary time-to-event outcomes were cancer-related survival, distant metastasis-free survival (DMFS), recurrence-free survival (RFS), disease-free survival (DFS) and breast cancer-specific survival (BCSS). Hazard ratios greater than 1 were oriented to represent worse outcomes with higher RAN. Published relative risks from survival analyses were retained using the terminology of the source and were not automatically relabelled as hazard ratios. Clinicopathological associations and marker-stratified findings were synthesised separately because they did not share a common effect definition.

The Liverpool nuclear RAN estimate was extracted from Yuen et al.’s original Cox regression, adjusted for tumour size, grade and nodal involvement [6]. The paper labels the exponentiated Cox effect as a relative risk (RR). In a Cox model, exp (beta) is a hazard ratio; this source terminology therefore denotes a relative-hazard estimate, not an odds ratio or a cumulative risk ratio derived from binary event counts. We retain “reported Cox RR” to preserve provenance. We did not reconstruct the protein estimate from a 2 × 2 table or independently refit the original patient data. An effect-measure audit is provided in Supplementary File S5.

### 2.5. Data Extraction and Cohort Overlap Assessment

One reviewer extracted study and outcome data using a piloted extraction template, and a second reviewer checked every entry against the source publication or dataset documentation. Discrepancies were resolved by consensus. Extracted variables included publication year, clinical setting, cohort size, recruitment source, assay, cellular localisation, expression threshold, endpoint, effect estimate, confidence interval, adjustment variables, and molecular or clinical strata. The complete blank extraction template is provided as Supplementary File S4. Institutional overlap was evaluated using sample size, specimen source, treatment eligibility and author/institution descriptions. Public data overlap was mapped by GEO accession. Within each forest plot, each institutional cohort or GEO accession contributed only one effect estimate. Endpoint-specific reuse of a cohort was allowed across separate analyses but never twice within the same analysis.

For primary reports authored by members of the review team, study-specific verification of eligibility, extraction, cohort overlap/effect selection, and risk-of-bias judgements was independently performed by two non-author reviewers (who were not authors of those reports). Details are given in Supplementary File S5.

The Liverpool family comprised Yuen 2012 [6], Yuen 2013 [7], Yuen 2016 [8], El-Tanani 2022 [10] and El-Tanani 2023 [11]. For the primary direct protein display, the adjusted nuclear RAN estimate from Yuen 2012 [6] was selected because it used the largest Liverpool tissue cohort and conventional clinicopathological adjustment. The cytoplasmic estimate from the same study and the untreated subset reported by El-Tanani 2022 [10] were reserved for replace-one sensitivity interpretation. The El-Tanani 2023 [11] report was not an additional independent protein estimate because it belongs to the same related Liverpool evidence family; its reported RR was retained for narrative interpretation. The primary protein display preserves each source’s Cox effect label and does not pool the two institutional estimates.

### 2.6. GEO Reanalysis

Unique GEO accessions were reanalysed separately. RAN was defined by the official gene symbol RAN and Entrez Gene 5901. Probe 200750_s_at was used on GPL96/GPL570, probe 18028 on GPL1223, and an outcome-independent mean z-score across probes 1839_at, 1840_g_at, and 38708_at on GPL8300. Raw-intensity-like data were log2-transformed; already normalised log-scale data were retained. The primary exposure compared expression at or above the within-accession median with expression below the median. A continuous effect per one within-accession standard deviation was examined as a sensitivity analysis.

Expression and endpoint fields were joined by GEO sample identifier and analysed as complete cases for each endpoint. A model required finite RAN expression, a finite non-negative follow-up time and a valid binary event indicator. No expression, survival or covariate values were imputed. GSE2034 follow-up and event data were taken from the archived nutshell version 1.0 GSE2034 clinical object and matched to the series matrix by GEO accession. The reanalysis used deposited series matrix annotations, including GSE1456, rather than substituting its separately updated clinical table. Supplementary File S5 provides the starting and final N, missing/invalid fields, non-overlapping total exclusions and event counts for every model, together with the public-sample-level analysis data and field mappings.

Univariable Cox proportional-hazards models with Efron handling of ties were fitted within each accession. DMFS included GSE2034, GSE7390 and GSE11121; RFS included GSE1379, GSE1456 and GSE7390; and OS included GSE1456, GSE3143 and GSE7390. The GEO datasets had been analysed or cited in the direct RAN literature; however, the cohort-specific HRs used in the present review were newly estimated from the accession-level clinical and RAN expression data using the harmonised analysis described above and were not extracted directly from the original publications. GSE1378 was excluded because it represents the same 60-patient Ma cohort as GSE1379. GSE4922 was analysed separately because its composite DFS endpoint included recurrence or breast cancer death. GSE12276 was excluded from Cox forests because all patients were selected for a known relapse site and no ordinary censoring/no-relapse indicator was available.

Endpoint assignment was checked against the actual deposited time and event fields and the available source documentation. The mappings were t.dmfs/e.dmfs for GSE11121 and GSE7390; months.to.relapse.or.last.followup/relapse for GSE2034 in its distant-relapse context; SURV_RELAPSE/RELAPSE for GSE1456; DFS with recur/non-recur status for GSE1379; SURV_DEATH/DEATH for GSE1456 OS; Surv(months)/STATUS for GSE3143; and t.os/e.os for GSE7390. GSE1379 therefore contributes a recurrence-coded DFS measure to the exploratory RFS family. GSE1456 BCSS uses DEATH_BC and was kept separate. The event definitions, coding, units, clinical context and unresolved details are listed in Supplementary File S5. Not all matrices fully specify recurrence components, time origins or competing-death/censoring rules, so identical endpoint definitions across cohorts cannot be assumed. The pools represent exploratory source-defined endpoint families.

### 2.7. Statistical Synthesis

For endpoints with three independent GEO estimates, log HRs were pooled using restricted maximum likelihood (REML) random-effects models with Hartung–Knapp confidence intervals [21]. Heterogeneity was quantified using τ^2^ and I^2^ [22]. Prediction intervals were calculated and are reported in Supplementary File S2; given the small number of cohorts, these were interpreted cautiously because of their substantial uncertainty. Accession-level Cox proportional-hazards models with Efron handling of ties were performed in Python 3.12.14, using NumPy 2.3.5, pandas 2.2.3, SciPy 1.17.0, and matplotlib 3.10.8 for data processing, statistical computation, and graphical output. The accession-level Efron models and REML/Hartung–Knapp pooling were reproduced in the Python environment, and all revised numerical outputs and forest plots were generated from that reproducible analysis. Cox standard errors used the observed information; meta-analytic confidence intervals used t(k − 1), and prediction intervals used t(k − 2) with the estimated between-cohort variance. The code, numerical outputs and data provenance are supplied with Supplementary File S5. The two independent protein expression estimates were displayed without a pooled summary because only two independent cohorts were available and between-study variation was substantial. Formal assessment of publication bias was not undertaken because each meta-analysis contained fewer than 10 independent estimates; funnel plots and regression-based asymmetry tests were therefore not performed. Meta-regression was also not undertaken. All subgroup analyses were exploratory; most were not prospectively prespecified, no multiplicity adjustment was applied, and the findings were interpreted as hypothesis-generating rather than clinically predictive.

## 3. Results

### 3.1. Study Selection

**Figure 1:**
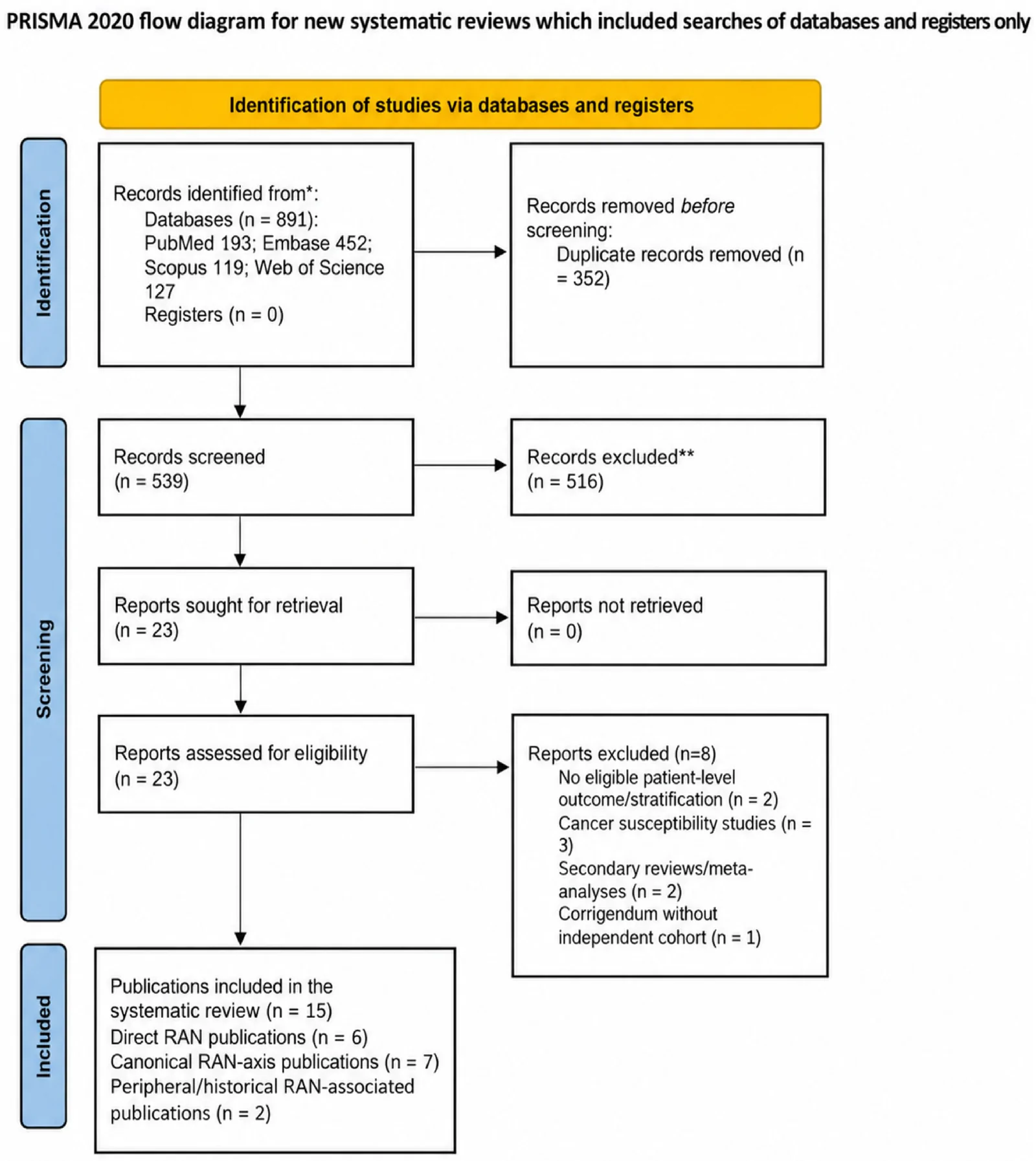
PRISMA 2020 study selection flow. Eight direct RAN and pathway extension exports yielded 891 records, 539 unique records after deduplication, 23 full-text reports assessed, and 15 included publications. The included publication count is distinct from the number of independent cohorts. All 23 sought reports were retrieved. * Records identified are reported separately for each database. ** Records were excluded during title and abstract screening by two independent reviewers. No automation tools were used for screening.

The eight exports contained 891 records: 193 from PubMed, 452 from Embase, 119 from Scopus, and 127 from Web of Science; 352 duplicate records were removed. The resulting 539 unique records were screened by title and abstract, and 516 were excluded. Twenty-three reports were sought, and all were retrieved for full-text assessment. Eight full-text reports were excluded: two lacked an eligible patient-level outcome or stratification analysis, three evaluated cancer susceptibility rather than outcomes after breast cancer diagnosis, two were secondary review/meta-analysis publications, and one was a corrigendum without an independent cohort. Fifteen clinical publications were retained [6,7,8,9,10,11,12,23,24,25,26,27,28,29,30]. The publication count should not be interpreted as an independent-cohort count. Two reviewers independently completed title/abstract and full-text screening, resolving disagreements by consensus. The study selection process is presented in Figure 1.

### 3.2. Study Characteristics and Evidence Structure

**Figure 2:**
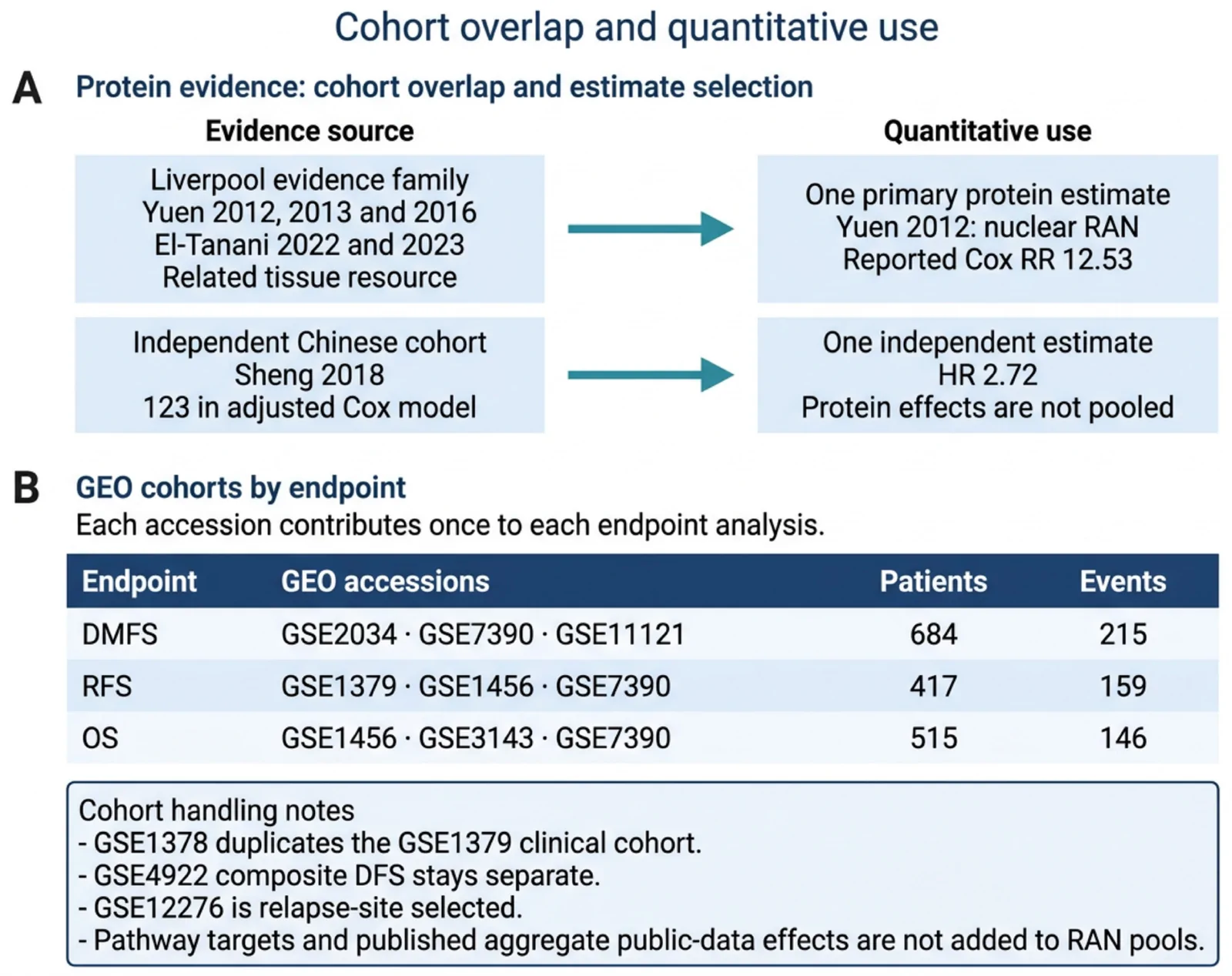
Cohort overlap and quantitative use of RAN evidence. (**A**) Protein-level evidence showing overlapping Liverpool publications by Yuen et al. [6,7,8] and El-Tanani et al. [10,11], from which one primary nuclear-RAN Cox estimate was selected, alongside the independent Chinese cohort reported by Sheng et al. [9]. The two protein estimates were displayed separately without pooling. (**B**) GEO cohorts included in the endpoint-specific analyses, showing the accession numbers, patient counts, and event counts for distant metastasis-free survival (DMFS), recurrence-free survival (RFS), and overall survival (OS). Each GEO accession contributed only once within each endpoint analysis to avoid double counting, although reuse across different endpoints was permitted. DFS, disease-free survival. Created in BioRender. Patni, M. A. (2026) https://BioRender.com/d72ftx7.

Fifteen publications met the clinical eligibility criteria. Six assessed direct RAN in tissue or transcriptomic cohorts [6,7,8,9,10,11]. Seven contributed canonical RAN-axis evidence: the broad pathway analysis by Vaidyanathan et al. [12], CSE1L/CAS [23], nucleocytoplasmic transport factors, including XPOT and CSE1L [25], XPOT in TNBC [27], RANBP3 methylation [28], XPOT in TCGA/METABRIC [29], and RANBP1 in TNBC [30]. Two reports were classified as peripheral or historical RAN-associated evidence: RANBP9/RanBPM [24] and the RANBP3 paralog RANBP3L [26]. These two studies were retained for contextual narrative synthesis only. Five direct RAN publications used the same or related Liverpool tissue resources, one independent Chinese institutional cohort evaluated RAN protein, and several reports reused public transcriptomic cohorts. Clinical evidence ranged from small diagnostic or tissue-localisation series to TCGA/METABRIC analyses; assays, thresholds and endpoints varied substantially. The characteristics and synthesis roles of the included publications are summarised in Table 1, and the cohort overlap structure is illustrated in Figure 2.

### 3.3. Direct RAN Protein Expression and Survival

Yuen et al. reported nuclear RAN positivity at a 1% staining cut-off to be associated with poorer patient survival in the Liverpool cohort (reported Cox RR 12.53, 95% CI 3.95–39.758; cohort n = 288) after adjustment for tumour size, grade and lymph-node status [6]. The original results explicitly identify Cox regression and refer to non-accessible Supplementary Files. A separate complete-case N and model event count could not be established from the available main report; the cohort size should not be assumed to be the fitted model size. Cytoplasmic RAN in the same resource was also adverse (reported Cox RR 2.50, 95% CI 1.59–3.93). These estimates were not entered together as independent observations.

Sheng et al. provided the principal independent institutional validation. Among 123 patients with complete multivariable data, high RAN protein expression was associated with poorer overall survival (HR 2.718, 95% CI 1.151–6.421) [9]. RAN was also higher in tumour than adjacent tissue and correlated strongly with Ki-67 (Spearman r = 0.834, *p* < 0.01). The magnitude of the Liverpool and Chinese estimates differed, but both pointed toward worse survival with high RAN. A pooled summary was withheld because only two independent tissue cohorts were available and the effect size difference was substantial. Repeated analyses of the Liverpool resource do not provide additional independent validation. These two cohorts are insufficient to establish a reproducible clinical prognostic model or incremental utility beyond established markers.

In the untreated Liverpool subset, El-Tanani et al. reported an adjusted RR of 3.69 (95% CI 1.09–12.49) for nuclear RAN [10]. This supports the direction of association but was treated as a replace-one sensitivity estimate rather than an additional cohort. The 2023 stage I–II study reported a high multivariable relative-risk estimate for nuclear RAN, but it was retained narratively and excluded as an additional independent estimate because of the related Liverpool cohort [11]. The direct RAN protein survival estimates and their quantitative-use decisions are summarised in Table 2.

### 3.4. RAN mRNA and Endpoint-Specific GEO Meta-Analysis

**Figure 3:**
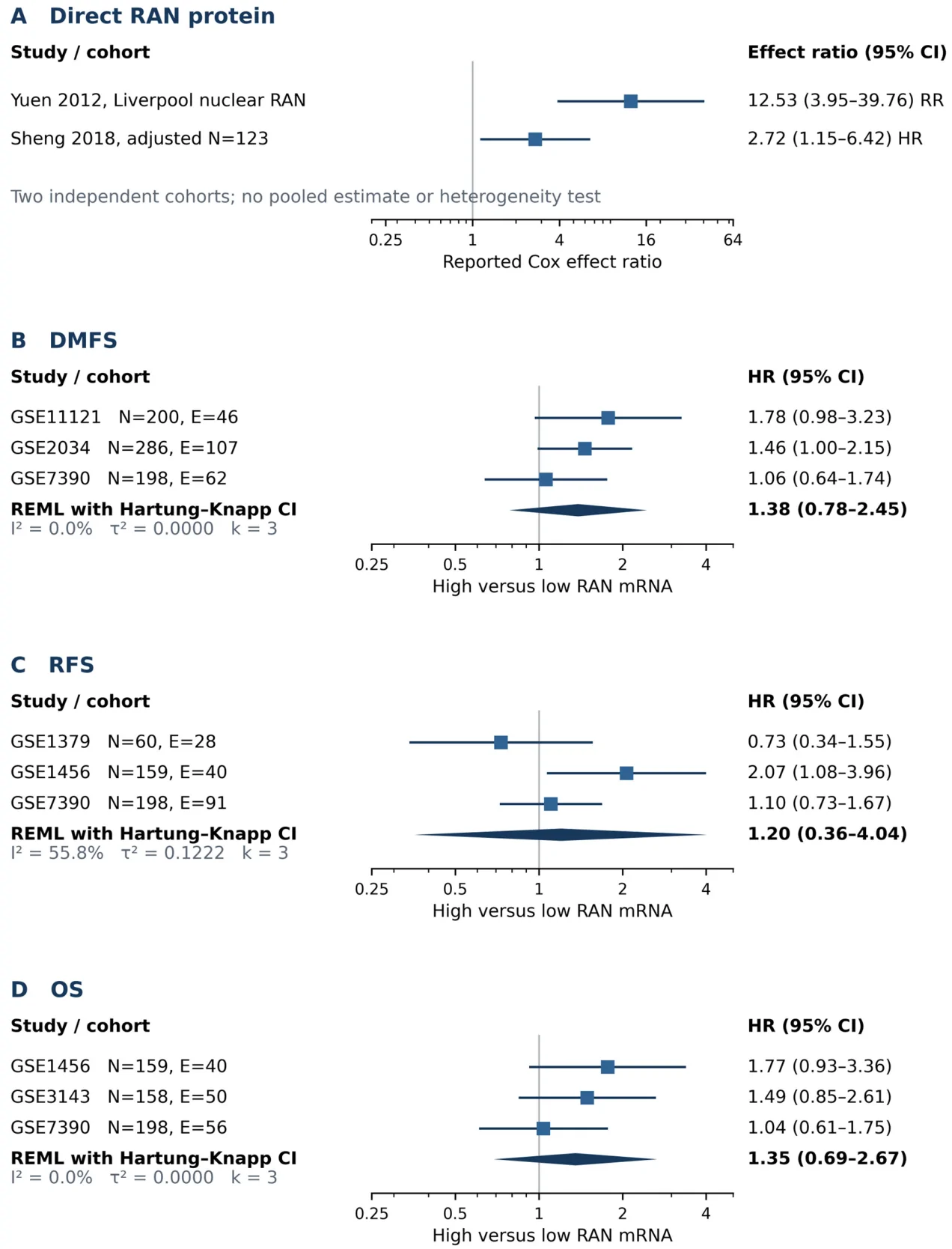
Direct RAN protein estimates and reverified accession-level RAN mRNA analyses. (**A**) Two independent institutional protein estimates from Yuen et al. [6] and Sheng et al. [9], with source terminology retained: the Liverpool effect is a reported Cox relative risk (RR), and Sheng et al. report a hazard ratio (HR). No pooled protein estimate or heterogeneity test is displayed. (**B**–**D**) Univariable Efron Cox HRs comparing RAN mRNA at or above the within-cohort median with values below the median for source-defined DMFS, RFS and OS families. N denotes patients in the Cox model, and E denotes observed events. Squares indicate individual cohort estimates; diamonds show REML random-effects estimates with Hartung–Knapp 95% confidence intervals. An effect above 1 indicates poorer outcome with higher RAN. Endpoint definitions are not assumed identical across cohorts; field mappings and exclusions are provided in Supplementary File S5. Prediction intervals are reported in Supplementary File S2.

The accession-level reanalysis prevented double-counting of patients across publications. For DMFS, the three-cohort pooled HR for high versus low RAN was 1.38 (95% CI 0.78–2.45; I-squared = 0%; tau-squared = 0). For RFS, the pooled HR was 1.20 (95% CI 0.36–4.04; I^2^ = 55.8%; τ^2^ = 0.1222). For OS, the pooled HR was 1.35 (95% CI 0.69–2.67; I-squared = 0%; tau-squared = 0). All the point estimates were adverse, but none was statistically conclusive under Hartung–Knapp inference. The prediction intervals are reported in Supplementary File S2 and were very wide, as expected with only three cohorts per endpoint. The moderate RFS heterogeneity may reflect differences among cohorts in patient composition, expression platforms, treatment exposure, endpoint definitions and sample size; however, with only three cohorts, these are possible rather than demonstrated explanations. The endpoint-specific pooled results are summarised in Table 3, and the direct protein and GEO analyses are displayed in Figure 3.

All nine primary accession-by-endpoint models retained every deposited sample with the required expression and outcome fields: DMFS included GSE11121 200/200 (46 events), GSE2034 286/286 (107 events) and GSE7390 198/198 (62 events); RFS included GSE1379 60/60 (28 events), GSE1456 159/159 (40 events) and GSE7390 198/198 (91 events); OS included GSE1456 159/159 (40 events), GSE3143 158/158 (50 events) and GSE7390 198/198 (56 events). Median and continuous models used the same patients. The GSE1456 BCSS sensitivity retained 159/159 (29 events). The separate GSE4922 composite DFS analysis excluded 40 of 289 samples lacking both time and event fields, leaving 249 patients and 89 events; none were excluded for missing RAN expression. These counts assess exclusions within the deposited series and do not rule out selection before deposition or informative missingness in GSE4922.

Continuous analyses per one standard deviation of RAN expression gave pooled HRs of 1.15 (95% CI 0.85–1.57) for DMFS, 1.20 (0.55–2.59) for RFS, and 1.20 (0.89–1.61) for OS. Proportional-hazards diagnostics flagged the continuous models in GSE11121 and GSE2034 and the median model in GSE3143, supporting cautious interpretation. In separate endpoint sensitivity analyses, high RAN was associated with worse composite DFS in GSE4922 (HR 1.68, 95% CI 1.10–2.57), while the GSE1456 BCSS estimate was adverse but imprecise (HR 2.01, 95% CI 0.94–4.33).

### 3.5. Molecular and Clinical Patient Stratification

All subgroup and paired-marker analyses in this section are exploratory. Most were not prospectively prespecified, no multiplicity adjustment was applied, and formal interaction testing was generally unavailable. Their findings are therefore hypothesis-generating and should not be interpreted as validated clinical prediction rules.

The observed prognostic association of RAN may vary with oncogenic context; this does not establish treatment response prediction. In Yuen 2012, the adverse effect of RAN was stronger in tumours with increased MET or osteopontin expression and in PIK3CA-mutant contexts [6]. In Yuen 2013, nuclear RAN was particularly adverse among MYC-positive tumours (reported RR 9.10, 95% CI 3.30–24.70), while transcriptomic effects were also larger in MYC-high than MYC-low groups [7]. These results support a biologically coherent RAN–MYC relationship but are not independent of the main Liverpool cohort.

Yuen 2016 further showed that MET predicted shorter survival clearly within RAN-high tumours (*p* < 0.001), whereas the association in RAN-low tumours was borderline (*p* = 0.052) [8]. El-Tanani 2022 identified a very high-risk RAN-positive/MMP2-positive group, with a reported RR of 82.1 and approximately 6% long-term survival [10]. The extreme magnitude and wide clinical separation are hypothesis-generating and require independent validation because the subgroup was derived from a modest, overlapping institutional cohort.

The stage I–II study suggested that nuclear RAN could refine conventional clinical classification. RAN positivity was adverse among node-negative patients (reported RR 13.17, 95% CI 3.18–54.58), ER-negative and ER-positive disease, HER2-negative disease, and triple-negative breast cancer (reported RR 19.81, 95% CI 4.67–83.99) [11]. These subgroup estimates indicate possible stratification value but should not be interpreted as validated treatment selection effects. The principal exploratory clinical and molecular stratification findings are summarised in Table 4.

### 3.6. RAN Pathways and Associated Clinical Evidence

**Figure 4:**
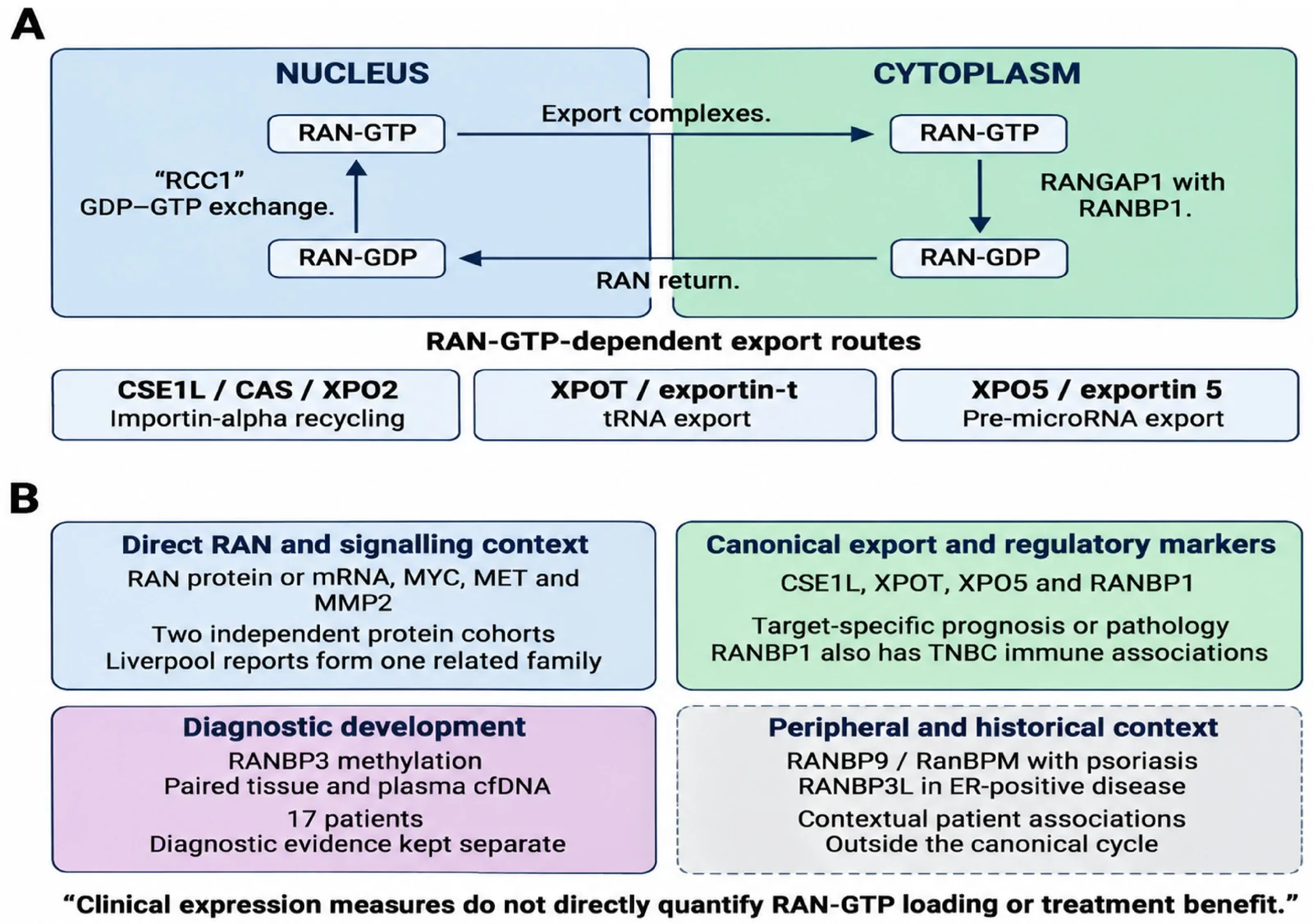
RAN pathways and the included clinical evidence. (**A**) Simplified RAN nucleotide cycle showing nuclear RCC1-mediated GDP–GTP exchange, cytoplasmic RANGAP1/RANBP1-mediated GTP hydrolysis, and representative CSE1L, XPOT, and XPO5 transport routes, based on previous studies [31,32,33,34]. Solid arrows indicate biological reactions or transport. (**B**) Overview of the clinical evidence, including direct RAN expression and signalling context [6,7,8,9,10,11], canonical export and regulatory markers [12,23,25,27,29,30], RANBP3 methylation in diagnostic development [28], and peripheral or historical RAN-associated markers [24,26]. RANBP9 and RANBP3L are presented separately from the canonical RAN cycle. Total RAN expression does not directly measure RAN-GTP loading, and observed patient associations do not establish treatment benefit. Background transport studies are not included in the 15 clinical publications. Additional publication mapping is provided in Supplementary File S6. TNBC, triple-negative breast cancer; cfDNA, cell-free DNA. Created in BioRender. Patni, M. A. (2026) https://BioRender.com/fchejz3.

The pathway map in Figure 4 separates established transport biology from the patient associations identified in this review. Its biological framework is supported by primary transport studies [31,32,33,34]; these background studies are not additional included clinical publications. The clinical evidence is organised below by transport/export function, RAN-binding regulation, oncogenic context and peripheral associations. The expression studies measured RAN protein or mRNA, which cannot be assumed to quantify the proportion of active, GTP-bound RAN.

#### 3.6.1. Core RAN Cycle and Functional Pathway Groups

Nuclear RCC1 promotes GDP–GTP exchange on RAN, whereas cytoplasmic RANGAP1, assisted by RANBP1, supports GTP hydrolysis and export-complex disassembly [31,32]. This spatial cycle coordinates cargo transport. Vaidyanathan et al. examined RAN and 16 associated components across public breast cancer datasets [12]. Nuclear export components showed the clearest associations with chromosome instability and adverse prognosis after clinical adjustment, whereas the import and mitotic spindle component groups did not show comparable prognostic associations in that analysis. This finding concerns expression-defined component groups and does not establish that export activity itself was measured in patients or that spindle functions are clinically irrelevant.

#### 3.6.2. Cargo Export and Importin Recycling

CSE1L/CAS, also termed exportin 2, recycles importin-alpha; XPOT/exportin-t transports tRNAs; and XPO5/exportin 5 transports pre-microRNAs [32,33,34]. These are distinct cargo routes rather than interchangeable RAN assays. In the clinical evidence, Vaidyanathan et al. identified CSE1L, XPOT, XPO5 and RANBP1 as adverse export-associated markers, with adjusted gene-specific HRs of approximately 1.21–1.33 [12]. Mehmood et al. also linked transport factor expression, including XPOT and CSE1L, with patient survival in public datasets [25]. Behrens et al. reported a shift in CSE1L localisation from weak, predominantly cytoplasmic staining in benign breast lesions to predominantly nuclear staining in invasive carcinomas [23]; this was a clinicopathological association rather than a survival estimate.

XPOT had additional target-specific patient evidence. Dai et al. associated higher XPOT with poorer survival in TNBC using a tissue microarray and public datasets [27]. Hou et al. linked XPOT expression with adverse overall survival and with ER-negative/PR-negative status, larger tumours, nodal involvement and basal subtype in TCGA/METABRIC analyses [29]. Their accompanying experimental work implicated selective tRNA export and TTC19 translation [27], or PI3K/AKT/mTOR signalling [29]. These mechanisms provide biological context; they do not establish that an XPOT assay predicts treatment benefit. Reuse of public cohorts and differences in assays and endpoints precluded additional target-specific pooling.

#### 3.6.3. RAN-Binding Regulators and Diagnostic Evidence

RANBP1 belongs to the transport regulatory arm of the RAN cycle. Beyond the export component analysis [12], Song et al. reported that high RANBP1 expression was associated with poorer survival and reduced T-cell infiltration in TNBC [30]. Their laboratory studies implicated a miR-769-5p/PRUNE2 mechanism, which remains distinct from a clinically validated immunotherapy response marker. RANBP3 contributed a different evidence type: Hu et al. examined tissue and plasma cfDNA methylation in 17 paired patients and reported diagnostic discrimination [28]. Methylation-based case discrimination is not a protein expression assay, a survival effect or evidence of clinical utility; this small diagnostic development study therefore remains separate from the prognostic synthesis.

#### 3.6.4. Oncogenic Signalling and Invasion Context

The direct RAN studies also examined MYC, MET and MMP2 contexts [6,7,8,10]. These are represented as signalling and invasion associations in Figure 4, with the patient-stratified results reported in Section 3.5. They are not independent pathway cohorts: the corresponding Liverpool publications belong to one related tissue family. The accompanying laboratory findings support mechanistic hypotheses, while the clinical strata remain exploratory associations without validated treatment-by-biomarker interactions.

#### 3.6.5. Peripheral and Historical Associations

Two publications sit outside the canonical transport map. In 64 invasive tumours, the psoriasin/RANBP9 (RanBPM) expression ratio was associated with ER-negative/PR-negative status and inflammatory infiltrates [24]. Higher RANBP3L protein expression in ER-positive disease was associated with lower histological grade [26]. RANBP9 and RANBP3L are retained as peripheral or historical context and are not placed in the RAN nucleotide cycle on the basis of nomenclature or paralogy. None of these target-specific results was pooled with direct RAN. Supplementary File S6 maps each publication to its pathway group, assay, patient stratum, outcome and synthesis role.

### 3.7. Risk of Bias and Certainty

**Figure 5:**
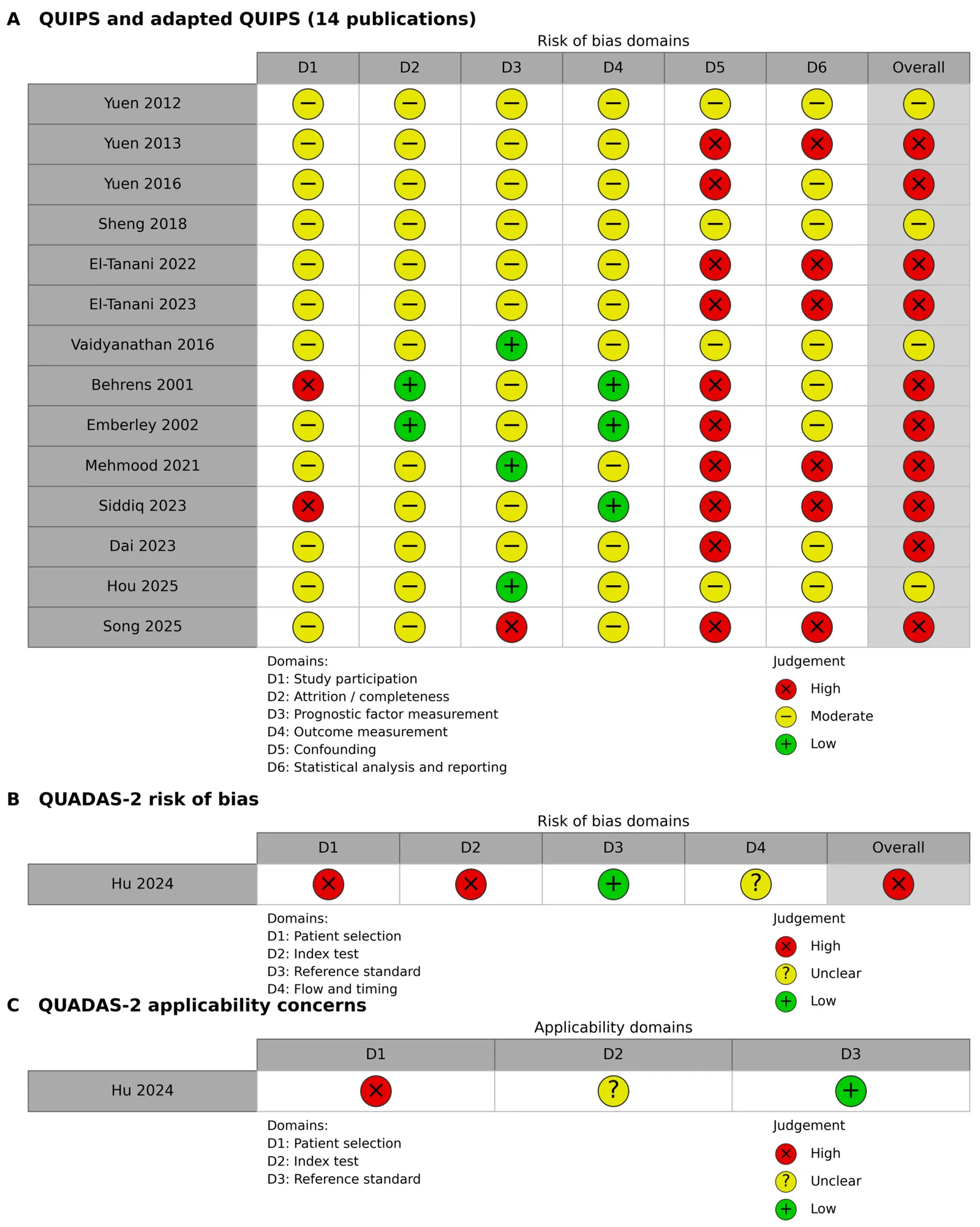
Design-specific risk-of-bias assessments. (**A**) Six-domain QUIPS or adapted QUIPS assessments for 14 prognostic or clinicopathological publications [6,7,8,9,10,11,12,23,24,25,26,27,29,30]. M denotes moderate risk, L denotes low risk, and H denotes high risk. “Completeness” represents attrition for prognostic cohorts and specimen/clinical-data completeness for cross-sectional studies. (**B**) Four QUADAS-2 risk-of-bias domains for the diagnostic study by Hu et al. [28]. (**C**) Three QUADAS-2 applicability domains for the same study [28]. A question mark denotes unclear risk or concern due to insufficient reporting. QUIPS and QUADAS-2 assessments are not combined into a numerical quality score. Domain-level rationales and adaptation criteria are provided in Supplementary File S3. Formal outcome-level certainty assessments are presented in Table 6 and Supplementary File S3. Certainty was low for the direct protein evidence, DMFS and OS, and very low for RFS and exploratory subgroup findings. The ratings reflect retrospective or early-phase evidence, risk of bias, sparse independent cohorts, imprecision, indirectness of legacy transcriptomic datasets and the absence of external validation. The current results support biomarker development rather than immediate clinical implementation.

Fourteen publications were assessed using QUIPS or adapted QUIPS: four were judged to have an overall moderate risk of bias and 10 a high risk; none met the conservative definition of overall low risk. The RANBP3 methylation diagnostic study was assessed separately using QUADAS-2 and was judged to have a high risk of bias, principally because only 17 paired patients were included, candidate methylation sites and thresholds were selected during marker development, and no independent diagnostic validation cohort was available. Thus, 10/14 QUIPS publications and 1/1 QUADAS-2 publication were high risk, giving a descriptive total of 11/15 across the separate tools. Domain-level judgements are presented in Figure 5A–C, while Table 5 summarises the overall study-level judgement, principal methodological concern, and implications for synthesis. Across the evidence base, the most frequent high-risk domains were confounding and statistical analysis/reporting. Recurrent concerns included selected retrospective cohorts, incomplete reporting of attrition and missing data, study-specific or outcome-optimised cut-offs, limited blinding information, multiple subgroup testing, small diagnostic or tissue series, and incomplete adjustment for established prognostic factors. Detailed domain-level rationales are provided in Supplementary File S3.

**Table 6:** Summary of findings and certainty of evidence.

Outcome	Evidence	Effect Estimate	Certainty	Reasons and Interpretation
Direct RAN protein and survival	2 independent cohorts; Liverpool cohort n = 288; Sheng adjusted model n = 123	RR 12.53 (3.95–39.76) and HR 2.72 (1.15–6.42); not pooled	Low	Only two retrospective institutional cohorts; moderate risk of bias, different assays and effect magnitudes, and imprecision. The Liverpool complete-case model N is not separately established. Prognostic magnitude and clinical utility remain uncertain.
DMFS	3 GEO cohorts; n = 684; 215 events	HR 1.38 (0.78–2.45); τ^2^ = 0; I^2^ = 0%	Low	Univariable legacy datasets, indirectness, imprecision and only three cohorts. Possible adverse association; statistically inconclusive.
RFS	3 GEO cohorts; n = 417; 159 events	HR 1.20 (0.36–4.04); τ^2^ = 0.1222; I^2^ = 55.8%	Very low	Risk of bias, moderate inconsistency, very serious imprecision and only three cohorts. Direction and magnitude remain highly uncertain.
OS	3 GEO cohorts; n = 515; 146 events	HR 1.35 (0.69–2.67); τ^2^ = 0; I^2^ = 0%	Low	Univariable legacy datasets, indirectness, imprecision and only three cohorts. Possible adverse association; statistically inconclusive.
Exploratory subgroup findings	Mostly overlapping Liverpool cohorts; variable subgroup sizes	Large heterogeneous reported effects; no pooled estimate	Very low	Mostly not prospectively prespecified, no multiplicity adjustment, sparse strata and incomplete interaction testing. Hypothesis-generating only; not clinically predictive.

## 4. Discussion

### 4.1. Principal Findings

This review identified a coherent but heterogeneous clinical literature linking elevated RAN expression and selected RAN-associated markers to adverse outcomes or clinically relevant strata in breast cancer. Direct protein expression studies still showed sizeable survival associations in the Liverpool and independent Chinese cohorts, while accession-level transcriptomic analyses produced adverse pooled point estimates for DMFS, RFS and OS with confidence intervals crossing the null. The added pathway reports strengthen biological and stratification context but do not increase the number of independent direct RAN estimates available for meta-analysis. The unusually large adjusted RR of 12.53 in Yuen 2012 should not be interpreted as a precise estimate of the true prognostic magnitude [6]. Sparse outcome events, instability in a multivariable model, selection of the biomarker cut-off, incomplete adjustment for contemporary prognostic variables, and limited external validation could all have inflated or destabilised this estimate.

The most clinically interesting signal is not a universal pan-breast cancer effect but the possibility that RAN identifies high-risk disease within defined biological contexts. MYC, MET and MMP2 may mark tumours in which RAN-associated biology is especially relevant, while node-negative, receptor-defined and triple-negative analyses suggest potential incremental prognostic information. Nevertheless, these exploratory findings largely arise from overlapping Liverpool specimens and require validation in external cohorts using prespecified assays and thresholds.

### 4.2. Biological Interpretation

RAN is positioned at the interface of nuclear transport and mitosis. Elevated RAN may support rapid proliferation by sustaining nucleocytoplasmic trafficking, spindle organisation and chromosome segregation [4,5]. The broader oncogenic roles of RAN across cancer hallmarks and therapeutic pathways have also been reviewed comprehensively [35]. Its association with MYC is plausible because MYC can transcriptionally regulate RAN, while RAN knockdown can reverse MYC-associated growth and invasion phenotypes [7]. MET-associated signalling may similarly increase reliance on RAN-mediated survival pathways [6,8]. The RAN–MMP2 relationship provides a link to extracellular-matrix remodelling, invasion and metastatic progression [10].

The nuclear export findings add an important nuance. Canonical RAN-axis evidence implicated CSE1L, XPOT, XPO5, RANBP1, and RANBP3 in target-specific prognostic or diagnostic analyses [12,23,25,27,28,29,30]. RANBP9/RanBPM and RANBP3L were classified separately as peripheral or historical markers because nomenclature or paralogy alone does not establish an equivalent RAN-dependent mechanism [24,26]. Some clinical consequences attributed broadly to RAN may arise through altered cargo transport or immune-microenvironment effects rather than spindle assembly alone. This creates opportunities for diagnosis, prognosis and target development, but also emphasises that direct RAN and individual pathway components are not interchangeable biomarkers.

### 4.3. Comparison with Other Prognostic Markers

RAN should be positioned alongside other nuclear transport candidates rather than regarded as a substitute for them. RAN is the guanine nucleotide switch coordinating transport and mitosis; XPO1/CRM1 is a cargo export receptor, RANBP1 regulates the RAN cycle, and CSE1L/CAS participates in importin-alpha recycling [4,12]. Their expression measurements therefore capture related but distinct processes. In a retrospective series of 280 breast cancers, higher XPO1/CRM1 expression was associated with shorter survival [36]. Within the studies included here, CSE1L localisation differed across benign and invasive lesions, while export-related transcriptomic analyses implicated CSE1L and RANBP1; the later RANBP1 study added TNBC-specific tissue and immune-microenvironment evidence [12,23,30]. These findings do not establish that any one marker is superior. No harmonised, externally validated head-to-head comparison of RAN with these candidates was identified in the included evidence. Differences in patients, assays, compartments, thresholds, endpoints, and adjustments prevent ranking markers by their reported effect sizes.

Ki-67 provides a particularly important comparator because it measures proliferation, a process closely related to RAN-associated biology. Sheng et al. reported a strong RAN-Ki-67 correlation (Spearman r = 0.834); RAN retained an association with survival in a multivariable model that included Ki-67 [9]. This is evidence of an adjusted association, but it does not demonstrate better discrimination, calibration or clinical utility than Ki-67. The correlation also raises the possibility that part of the RAN signal reflects a shared proliferative programme. The International Ki67 Working Group emphasises standardised specimen handling, calibrated scoring and quality assurance, with clinical utility confined to defined settings [37]. A RAN assay requires comparable analytical standardisation and external testing of the information it adds after accounting for proliferation.

The gap in clinical validation is larger when RAN is compared with multigene assays. The 21-gene recurrence score and the 70-gene signature have been evaluated in prospective randomised trials addressing adjuvant chemotherapy decisions [38,39,40]. TAILORx studied hormone-receptor-positive, HER2-negative, node-negative disease; RxPONDER studied patients with one to three positive nodes and recurrence scores of 25 or lower, with chemotherapy findings differing by menopausal status [38,39]. MINDACT evaluated the consequences of discordance between clinical and genomic risk [40]. Their evidence is specific to the populations, treatments and risk ranges studied, and should not be generalised to every breast cancer subtype. Nevertheless, these trials provide decision-oriented evidence that is not available for RAN. Neither an adverse RAN association nor a large retrospective effect estimate establishes equivalence to a genomic assay or predicts chemotherapy benefit.

The relevant development question is therefore whether RAN improves a specified prognostic model beyond stage, grade, nodal status, ER, PR, HER2, Ki-67 and treatment, and beyond a validated genomic score when appropriate to the intended population. This requires comparison within the same patients using a fixed outcome and time horizon, rather than indirect comparison of hazard ratios from unrelated cohorts. Until such incremental value and reproducibility are demonstrated, RAN remains a candidate adjunct for biomarker development and cannot replace established pathological assessment or validated genomic testing.

### 4.4. Clinical Implications

A clinically useful RAN assay would need to provide information beyond established variables and to do so reproducibly. The strongest current evidence concerns prognosis, not prediction of benefit from a particular therapy. Therefore, high RAN should not yet be used to select treatment. A development pathway should first standardise specimen handling, antibody clone, cellular compartment, scoring system and cut-off. The assay should then be tested in independent, contemporary cohorts with multivariable models incorporating stage, grade, nodes, ER, PR, HER2, Ki-67 and treatment. A prognostic marker identifies differences in outcome risk. Predictive treatment value requires evidence that a treatment effect differs by marker status, supported by an appropriate treatment-by-RAN interaction in comparative treatment data and subsequent independent validation. Neither a survival association in a treated cohort nor preclinical sensitivity to RAN inhibition establishes this.

The most efficient validation strategy may be clinically enriched rather than purely pan-cohort. Prespecified analyses could focus on node-negative disease, triple-negative breast cancer and tumours with high MYC, MET or MMP2. However, predictive claims require a treatment-by-biomarker interaction or a biomarker-guided trial; an association with poor outcome in a treated group is not sufficient. Prospective studies should also determine whether nuclear, cytoplasmic or total RAN is the most reproducible clinical measurement.

### 4.5. Candidate RAN Panels and Their Validation

Two complementary panel strategies merit investigation. A pragmatic prognostic panel could add a prespecified nuclear or cytoplasmic RAN score to an established clinicopathological model, including receptor status and Ki-67. The IHC4 study illustrates the principle of combining ER, PR, HER2 and Ki-67 and comparing the resulting score with a genomic recurrence score; it did not evaluate RAN [41]. A RAN extension would need to improve on the baseline model and on RAN alone, especially because RAN and Ki-67 are strongly correlated. In ER-positive/HER2-negative settings where genomic testing is relevant, any added value should also be tested against the applicable genomic score rather than assumed from an adjusted RAN association.

A second strategy could examine biological context using RAN with MYC, MET or MMP2, or a small transport-focused set containing RAN with XPO1, RANBP1 or CSE1L [7,8,10,12,30,36]. These are candidate combinations, not validated panels. The existing joint-marker and subgroup findings largely reuse Liverpool specimens and cannot supply independent validation. A significant association in one marker-defined subgroup and a non-significant association in another do not, by themselves, demonstrate interaction or biological synergy. Highly correlated transport markers could also add redundant information, so a panel should remain parsimonious and its contribution should be compared with that of RAN alone.

Panel development should specify the intended patient group, endpoint and prediction horizon, assay compartments, scoring rules and candidate variables before examining outcomes. Continuous measurements should be retained where appropriate, with any clinical thresholds fixed before external validation. Model complexity should be limited by the available outcome events; penalisation and bootstrap validation can reduce overfitting, but cannot replace evaluation of a locked model in independent contemporary cohorts. Baseline and extended models should be compared for discrimination, calibration and clinical net benefit at relevant decision thresholds, with uncertainty reported, and multiplicity addressed [18]. Patient overlap must be excluded across development and validation samples. These proposals are hypothesis-generating; treatment selection claims would additionally require a supported treatment-by-marker interaction and evidence that using the panel improves decisions or outcomes.

### 4.6. Methodological Strengths and Limitations

The principal strength of this review was explicit management of cohort overlap. By treating the cohort or accession as the statistical unit, the analysis avoided counting repeated Liverpool specimens and GEO patients as independent evidence. The GEO analyses applied an outcome-independent probe and cut-off rule and kept the source-defined DMFS, RFS and OS families separate from composite DFS and BCSS. Complete harmonisation of endpoint components, time origins and censoring conventions was not possible from the deposited annotations. This is more defensible than pooling published aggregate effects that reuse the same patients.

Several limitations remain. Although the search yielded 15 eligible clinical publications, five direct RAN reports belonged to one related institutional evidence family, and the additional reports evaluated different pathway targets. The review protocol is registered on the Open Science Framework (https://doi.org/10.17605/OSF.IO/BV3NG). No included publication was judged overall low risk of bias; 10 of 14 QUIPS/adapted-QUIPS publications were high risk and four moderate; the separate QUADAS-2 publication was high risk, giving 11 of 15 high-risk publications overall, driven mainly by confounding, small selected cohorts, outcome-optimised biomarker thresholds, multiple exploratory subgroup analyses and incomplete statistical reporting. Some publications combined clinical and laboratory objectives, so patient selection and outcome reporting were not optimised for prognostic validation. IHC methods and positivity thresholds differed, and some source Cox estimates were labelled relative risks; these source labels were retained rather than treated as cumulative risk ratios. The direct RAN protein evidence rested on only two independent retrospective cohorts and was too sparse for a stable pooled estimate or a claim of validated prognostic utility. GEO meta-analyses included only three cohorts per endpoint, making heterogeneity and prediction intervals imprecise. The moderate RFS heterogeneity may be related to cohort composition, assay platforms, treatments, endpoint definitions or sample sizes, but these explanations could not be tested and should not be regarded as proven. Publication bias could not be formally assessed because each meta-analysis contained fewer than 10 independent estimates. Most subgroup analyses were post hoc or not prospectively prespecified, no multiplicity correction was applied, and interaction testing was generally absent; consequently, they are hypothesis-generating rather than clinically predictive. Several contextual studies reused TCGA, METABRIC or GEO data, and no target was pooled across overlapping public datasets. Finally, several eligible primary reports were authored by members of the review team.

### 4.7. Research Priorities

Future work should prioritise: (1) independent multicentre validation of nuclear and cytoplasmic RAN protein; (2) a prespecified, analytically validated IHC scoring method; (3) external validation of MYC-, MET- and MMP2-based joint-marker models; (4) assessment of incremental value beyond clinicopathological and genomic predictors; (5) transparent publication of complete Cox models and subgroup interaction tests; and (6) prospective studies linking biomarker status to treatment response. Public data studies should publish accession-level effects and avoid presenting overlapping datasets as independent replications.

## 5. Conclusions

Elevated tumour RAN expression was associated with adverse survival in two independent institutional cohorts, while transcriptomic meta-analyses produced adverse but statistically inconclusive pooled estimates. The expanded systematic review also identified target-specific canonical evidence for CSE1L, RANBP1, RANBP3, XPOT and XPO5. RANBP9/RanBPM and RANBP3L provided peripheral contextual evidence only. All pathway marker findings must remain separate from the direct RAN meta-analysis until independent, comparable cohorts are available for each individual target.

The evidence is insufficient for routine clinical diagnosis, prognostic classification or treatment selection. The next decisive step is prospective, independent validation using standardised assays, prespecified cut-offs and multivariable models, with explicit testing of whether RAN adds clinically meaningful information beyond established breast cancer markers. No included association establishes that RAN predicts benefit from a particular treatment, and the proposed strata must not be used to select therapy.

## Figures and Tables

**Table 1:** Characteristics and analytical role of the 15 included clinical publications.

Study	Clinical Evidence	RAN Assessment	Outcome	Target or Stratum	Tier|Synthesis Role
Yuen 2012 [6]	Liverpool IHC n = 288; GEO n = 564	Nuclear/cytoplasmic protein; mRNA	Patient survival	MET, OPN, PIK3CA	Direct RAN|primary Liverpool Cox RR
Yuen 2013 [7]	Liverpool n = 288; GEO n = 1454	Nuclear/cytoplasmic protein; mRNA	Patient survival	MYC	Direct RAN|MYC strata
Yuen 2016 [8]	Liverpool specimens n = 247	Nuclear, cytoplasmic and total protein	Patient survival	MET	Direct RAN|RAN–MET strata
Sheng 2018 [9]	Independent Chinese cohort n = 140; Cox n = 123	RAN protein by IHC	Overall survival	Ki-67; grade; invasion	Direct RAN|independent validation
El-Tanani 2022 [10]	Untreated Liverpool subset n = 181	Nuclear RAN and MMP2 protein	Cancer-related survival	MMP2, MET, MYC	Direct RAN|RAN-MMP2 strata
El-Tanani 2023 [11]	Liverpool n = 263; public validation n = 1079	RAN protein and mRNA	Survival, DMFS, RFS	Nodes, ER, HER2, TNBC	Direct RAN|clinical strata
Vaidyanathan 2016 [12]	Twenty-six public datasets; maximum n = 4177	RAN and 16 pathway mRNAs	Metastatic relapse/DMFS	Nuclear export targets	Canonical axis|pathway discovery
Behrens 2001 [23]	Breast lesions n = 50 (13 benign, 17 in situ, 20 invasive)	CSE1L/CAS IHC and localisation	Invasive status	Benign, in situ, invasive	Canonical axis|CSE1L localisation
Emberley 2002 [24]	Invasive tumours n = 64	RANBP9/RanBPM and psoriasin mRNA	ER, PR, inflammatory infiltrate	Psoriasin/RanBPM ratio	Peripheral/historical|RanBPM context
Mehmood 2021 [25]	TCGA and public microarray cohorts	Nucleocytoplasmic transport genes	Patient survival	Transport factor expression	Canonical axis|public data context
Siddig 2023 [26]	ER-positive discovery n = 13; RANBP3L IHC n = 74	RANBP3L mRNA/protein	Grade and age group	Young vs. older; tumour grade	Peripheral/historical|paralog context
Dai 2023 [27]	TNBC tissue microarray n = 80 plus TCGA/GEO	XPOT protein and mRNA	Overall survival; tumour features	High vs. low XPOT	Canonical axis|XPOT prognosis
Hu 2024 [28]	Paired tissue/plasma from n = 17	RANBP3 methylation	Diagnostic discrimination; subtype	Tumour vs. adjacent; HR status	Canonical axis|RANBP3 diagnosis
Hou 2025 [29]	TCGA n = 966; METABRIC n = 1904	XPOT mRNA	Overall survival; clinicopathology	Receptor status, size, nodes, subtype	Canonical axis|XPOT prognosis
Song 2025 [30]	TNBC IHC n = 87; TCGA/GEO/scRNA-seq	RANBP1 protein and mRNA	Overall survival; immune infiltration	High vs. low RANBP1	Canonical axis|RANBP1 prognosis

**Table 2:** Direct RAN survival estimates and quantitative-use decisions.

Study/Cohort	N	Outcome	Estimate (95% CI)	Adjustment	Decision
Yuen 2012 [6], nuclear RAN	288 (cohort)	Patient survival	Reported Cox RR 12.53 (3.95–39.76)	Size, grade, nodes	Primary Liverpool estimate
Yuen 2012 [6], cytoplasmic RAN	288 (cohort)	Patient survival	Reported Cox RR 2.50 (1.59–3.93)	Size, grade, nodes	Localisation sensitivity
Sheng 2018	123 (model)	Overall survival	HR 2.718 (1.151–6.421)	Multivariable Cox	Independent primary estimate
El-Tanani 2022 [10]	181	Cancer-related survival	RR 3.69 (1.09–12.49)	MET, MYC, MMP2	Replace-one sensitivity
El-Tanani 2023 [11]	263	Patient survival	Reported RR 20.16 (2.78–146.22)	Multivariable model	Narrative

**Table 3:** Endpoint-specific meta-analysis of unique GEO cohorts.

Endpoint	Cohorts	k	Pooled HR (95% CI)	I-Squared	τ^2^	Interpretation
DMFS	GSE2034, GSE7390, GSE11121	3	1.38 (0.78–2.45)	0%	0.0000	Not statistically conclusive
RFS	GSE1379, GSE1456, GSE7390	3	1.20 (0.36–4.04)	55.8%	0.1222	Not statistically conclusive
OS	GSE1456, GSE3143, GSE7390	3	1.35 (0.69–2.67)	0%	0.0000	Not statistically conclusive

**Table 4:** Principal clinical and molecular stratification findings.

Study	Marker Context	Patient Stratum	Result	Clinical Interpretation
Yuen 2012 [6]	RAN with MET/OPN/PIK3CA	Pathway-activated tumours	Stronger adverse RAN association	Oncogenic context may identify RAN dependence
Yuen 2013 [7]	RAN with MYC	MYC-positive tumours	Nuclear RAN RR 9.10 (3.30–24.70)	MYC may refine prognostic use of RAN
Yuen 2016 [8]	MET within RAN groups	RAN-high tumours	MET *p* < 0.001; RAN-low *p* = 0.052	Supports a RAN–MET interaction hypothesis
El-Tanani 2022 [10]	RAN with MMP2	Double-positive tumours	Reported RR 82.1	Very-high-risk subgroup; independent validation needed
El-Tanani 2023 [11]	RAN with clinical markers	Node-negative, ER, HER2-negative, TNBC	Consistently adverse subgroup estimates	Potential refinement of conventional risk groups
Vaidyanathan 2016 [12]	Nuclear export signature	Public transcriptomic cohorts	Adjusted HR 1.16 (1.01–1.34)	Target discovery evidence

**Table 5:** Study-level risk-of-bias judgements and implications for synthesis.

Study	Tool	Overall	Principal Concern	Synthesis Handling
Yuen 2012 [6]	QUIPS	Moderate	Retrospective cohort; incomplete blinding, missing data and cut-off reporting.	Primary Liverpool estimate retained.
Yuen 2013 [7]	QUIPS	High	Limited confounder control, repeated subgroup analyses and overlapping public datasets.	MYC-stratified narrative only.
Yuen 2016 [8]	QUIPS	High	Incomplete adjustment and no formal interaction estimate for RAN–MET strata.	RAN–MET narrative only.
Sheng 2018 [9]	QUIPS	Moderate	Single-centre cohort; incomplete-case and IHC-threshold reporting.	Independent primary estimate retained.
El-Tanani 2022 [10]	QUIPS	High	Overlapping subset, sparse joint-marker groups and potentially unstable large effects.	Replace-one sensitivity; narrative strata.
El-Tanani 2023 [11]	QUIPS	High	Multiple strata, metric ambiguity and overlapping Liverpool/GEO data.	No pooling
Vaidyanathan 2016 [12]	QUIPS	Moderate	Heterogeneous public datasets; adjusted analysis available only for a subset.	Target discovery/sensitivity only.
Behrens 2001 [23]	Adapted QUIPS	High	Small selected pathology series without adjusted analysis.	CSE1L localisation narrative.
Emberley 2002 [24]	Adapted QUIPS	High	Selected tumours, study-specific ratio/cut-off and no confounder adjustment.	Tier C context only.
Mehmood 2021 [25]	QUIPS	High	Exploratory multigene screening, multiple testing and limited clinical adjustment.	Public data narrative only.
Siddig 2023 [26]	Adapted QUIPS	High	Very small discovery cohort and incompletely adjusted exploratory comparisons.	Tier C context only.
Dai 2023 [27]	QUIPS	High	Small TNBC tissue series, incomplete adjustment and reused public cohorts.	XPOT narrative evidence.
Hou 2025 [29]	QUIPS	Moderate	Retrospective TCGA/METABRIC analysis with residual confounding and overlap risk.	XPOT evidence; deduplicate datasets.
Song 2025 [30]	QUIPS	High	X-tile cut-off, baseline imbalance and inadequate adjusted prognostic modelling.	RANBP1 hypothesis-generating only.
Hu 2024 [28]	QUADAS-2	High	Only 17 patients; development-stage threshold selection and no external validation.	Diagnostic narrative only.

## Data Availability

The complete database-specific search strategies, yields, deduplication rules, PRISMA counts, full-text exclusion log and included-study classification are provided in Supplementary File S1. Quantitative estimates, cohort overlap decisions and prediction intervals are provided in Supplementary File S2. Domain-level QUIPS and QUADAS-2 judgements, and the certainty assessment are provided in Supplementary File S3. The piloted extraction template is provided in Supplementary File S4. Evidence-tier classification, model-level sample-flow and endpoint audits, public-sample-level GEO analysis data, source effect-measure checks, risk-of-bias denominators, and the non-author verification record are supplied in Supplementary File S5. The pathway diagram and publication-level clinical and biological source mapping are supplied in Supplementary File S6.

## Supplementary Materials

The following supporting information can be downloaded at: https://www.mdpi.com/article/10.3390/cancers18213591/s1, Supporting information referred to in this manuscript comprises Supplementary File S1 (database-specific search strategies, study selection records and evidence classification); Supplementary File S2 (quantitative estimates, cohort overlap decisions and prediction intervals); Supplementary File S3 (QUIPS, QUADAS-2 and certainty assessments); Supplementary File S4 (blank piloted data-extraction template); Supplementary File S5 (GEO reanalysis data, endpoint and effect-measure audits, and independent-verification records); and Supplementary File S6 (pathway mapping and source classification).
