# Supplementary material for: Prognostic Significance of RAN Expression and RAN-Associated Pathways in Breast Cancer: A Systematic Review and Exploratory Meta-Analysis
Source: Cancers (Basel). 2026 Nov 9;18(21):3591. doi: 10.3390/cancers18213591 (PMC13761802; doi:10.3390/cancers18213591)
Supplement: Supplementary file 1 [file cancers-18-03591-s001.zip › Final_Supplementary_File_S1.pdf]

Supplementary File S1. Search and Selection

Prognostic Significance of RAN Expression and RAN-Associated Pathways in Breast Cancer: A Systematic Review and Exploratory Meta-analysis

Databases: PubMed, Embase, Scopus and Web of Science Core Collection

Searches: one direct-RAN search and one pathway-extension search per database; no date, language or study-design filters were applied.

S1. Information sources and search principles

The database search was designed for sensitivity. The direct-RAN strategy did not require an outcome term because survival and patient-stratification findings were frequently secondary clinical analyses and were not consistently indexed in titles or abstracts. A separate pathway-extension strategy targeted predefined RAN-associated nuclear-transport markers that could provide clinically relevant diagnostic, prognostic or clinicopathological stratification evidence. The isolated term RAN generated predictable noise, including the ordinary verb “ran”, author names and ranolazine; these records were removed during screening rather than by an outcome filter.

S2. Exact database-specific search strategies

PubMed — direct RAN

Yield exported: n=186.

```
(
  "ran GTP-binding protein"[MeSH Terms]
  OR "RAN GTPase"[Title/Abstract]
  OR RanGTPase[Title/Abstract]
  OR "Ran-GTPase"[Title/Abstract]
  OR "Ras-related nuclear protein"[Title/Abstract]
  OR "GTP-binding nuclear protein Ran"[Title/Abstract]
  OR "RAN protein"[Title/Abstract]
  OR "RAN expression"[Title/Abstract]
  OR RAN[Title/Abstract]
)
AND
(
  "Breast Neoplasms"[MeSH Terms]
  OR "breast cancer"[Title/Abstract]
  OR "breast cancers"[Title/Abstract]
  OR "breast carcinoma"[Title/Abstract]
  OR "breast carcinomas"[Title/Abstract]
  OR "breast tumor"[Title/Abstract]
  OR "breast tumour"[Title/Abstract]
  OR "mammary carcinoma"[Title/Abstract]
  OR "mammary cancer"[Title/Abstract]
)
```

PubMed — pathway extension

Yield exported: n=7.

```
(
  RANBP1[Title/Abstract] OR RANBP3[Title/Abstract] OR RANBP3L[Title/Abstract]
  OR RANBP9[Title/Abstract] OR RanBPM[Title/Abstract]
  OR CSE1L[Title/Abstract] OR "cellular apoptosis susceptibility"[Title/Abstract]
  OR XPOT[Title/Abstract] OR "exportin-T"[Title/Abstract]
  OR XPO5[Title/Abstract] OR "exportin 5"[Title/Abstract]
)
AND
(
  "Breast Neoplasms"[MeSH Terms]
  OR "breast cancer"[Title/Abstract]
  OR "breast carcinoma"[Title/Abstract]
  OR "breast tumor"[Title/Abstract]
  OR "breast tumour"[Title/Abstract]
  OR "mammary carcinoma"[Title/Abstract]
)
```

Embase — direct RAN

Yield exported: n=438.

```
('ran gtp binding protein'/exp OR 'ran gtpase':ti,ab,kw OR rangtpase:ti,ab,kw
 OR 'ran-gtpase':ti,ab,kw OR 'ras related nuclear protein':ti,ab,kw
 OR 'gtp binding nuclear protein ran':ti,ab,kw OR 'ran protein':ti,ab,kw
 OR 'ran expression':ti,ab,kw OR ran:ti,ab,kw)
AND
('breast tumor'/exp OR 'breast cancer':ti,ab,kw OR 'breast carcinoma':ti,ab,kw
 OR 'breast tumour':ti,ab,kw OR 'breast tumor':ti,ab,kw
 OR 'mammary carcinoma':ti,ab,kw OR 'mammary cancer':ti,ab,kw)
```

Embase — pathway extension

Yield exported: n=14.

```
(ranbp1:ti,ab,kw OR ranbp3:ti,ab,kw OR ranbp3l:ti,ab,kw
 OR ranbp9:ti,ab,kw OR ranbpm:ti,ab,kw OR cse1l:ti,ab,kw
 OR 'cellular apoptosis susceptibility':ti,ab,kw OR xpot:ti,ab,kw
 OR 'exportin t':ti,ab,kw OR xpo5:ti,ab,kw OR 'exportin 5':ti,ab,kw)
AND
('breast tumor'/exp OR 'breast cancer':ti,ab,kw OR 'breast carcinoma':ti,ab,kw
 OR 'breast tumour':ti,ab,kw OR 'breast tumor':ti,ab,kw
 OR 'mammary carcinoma':ti,ab,kw)
```

Scopus — direct RAN

Yield exported: n=103.

```
TITLE-ABS-KEY(
  "RAN GTPase" OR RanGTPase OR "Ran-GTPase" OR "Ras-related nuclear protein"
  OR "GTP-binding nuclear protein Ran" OR "RAN protein" OR "RAN expression" OR RAN
)
AND TITLE-ABS-KEY(
  "breast cancer" OR "breast cancers" OR "breast carcinoma" OR "breast carcinomas"
  OR "breast tumor" OR "breast tumour" OR "mammary carcinoma" OR "mammary cancer"
)
```

Scopus — pathway extension

Yield exported: n=16.

```
TITLE-ABS-KEY(
  RANBP1 OR RANBP3 OR RANBP3L OR RANBP9 OR RanBPM OR CSE1L
  OR "cellular apoptosis susceptibility" OR XPOT OR "exportin-T" OR XPO5 OR "exportin 5"
)
AND TITLE-ABS-KEY(
  "breast cancer" OR "breast carcinoma" OR "breast tumor" OR "breast tumour"
  OR "mammary carcinoma"
)
```

Web of Science Core Collection — direct RAN

Yield exported: n=112.

```
TS=(
  "RAN GTPase" OR RanGTPase OR "Ran-GTPase" OR "Ras-related nuclear protein"
  OR "GTP-binding nuclear protein Ran" OR "RAN protein" OR "RAN expression" OR RAN
)
AND TS=(
  "breast cancer" OR "breast cancers" OR "breast carcinoma" OR "breast carcinomas"
  OR "breast tumor" OR "breast tumour" OR "mammary carcinoma" OR "mammary cancer"
)
```

Web of Science Core Collection — pathway extension

Yield exported: n=15.

```
TS=(
  RANBP1 OR RANBP3 OR RANBP3L OR RANBP9 OR RanBPM OR CSE1L
  OR "cellular apoptosis susceptibility" OR XPOT OR "exportin-T" OR XPO5 OR "exportin 5"
)
AND TS=(
  "breast cancer" OR "breast carcinoma" OR "breast tumor" OR "breast tumour"
  OR "mammary carcinoma"
)
```

S3. Database yields

Database	Direct-RAN search	Pathway extension	Total exported
PubMed	186	7	193
Embase	438	14	452
Scopus	103	16	119
Web of Science	112	15	127
Total	839	52	891

S4. Deduplication and screening workflow

PRISMA 2020 stage	Count
Records identified from databases	891
Duplicate records removed	352
Unique records screened	539
Records excluded at title/abstract	516
Reports sought for retrieval	23
Reports not retrieved	0
Full-text reports assessed	23
Full-text reports excluded	8
Clinical publications included	15

S5. Eligibility criteria applied during screening

- Population: patients with breast cancer or human breast tumour specimens/datasets with clinical annotation.
- Core exposure: direct tumour RAN protein or RAN mRNA expression.
- Contextual exposure: a predefined RAN-associated marker (RANBP1, RANBP3, RANBP3L, RANBP9/RanBPM, CSE1L/CAS, XPOT or XPO5) with an established RAN-axis/nuclear-transport role.
- Eligible outcomes: time-to-event prognosis, diagnostic discrimination, receptor or molecular subtype, stage, grade, nodal status, invasion/metastasis or another patient-level clinicopathological stratification result.
- Eligible designs: clinical tissue cohorts, diagnostic paired-sample studies and secondary analyses of annotated public patient cohorts.

- Exclusions: no original human breast-cancer data; no eligible patient-level outcome; cell/animal/in-silico evidence alone; conference abstract without sufficient full data; review/editorial; non-breast cancer; germline susceptibility study comparing cases with cancer-free controls; duplicate or corrigendum without an independent cohort.

S6. Full-text exclusion log

Report	Title	Primary exclusion reason
Jang et al., 2020	Computational inference of cancer-specific vulnerabilities in clinical samples	No eligible patient-level survival, diagnostic or clinicopathological stratification outcome
Hansen et al., 2001	Identification of immunogenic antigens using a phage-displayed cDNA library from an invasive ductal breast carcinoma tumour	No eligible patient-level outcome/stratification analysis
Jiang et al., 2013	Evaluation of genetic variants in microRNA biosynthesis genes and risk of breast cancer in Chinese women	Cancer susceptibility rather than outcome after breast-cancer diagnosis
Bermisheva et al., 2018	MicroRNA biogenesis pathway gene polymorphisms are associated with breast cancer risk	Cancer susceptibility rather than outcome after breast-cancer diagnosis
Sung et al., 2011	Common genetic polymorphisms of microRNA biogenesis pathway genes and risk of breast cancer: a case-control study in Korea	Cancer susceptibility rather than outcome after breast-cancer diagnosis
Elshazli et al., 2023	Pan-cancer study on variants of canonical miRNA biogenesis pathway components: a pooled analysis	Secondary pooled analysis; no independent primary cohort
Shao et al., 2020	Association of microRNA biosynthesis genes XPO5 and RAN polymorphisms with cancer susceptibility: Bayesian hierarchical meta-analysis	Secondary meta-analysis
El-Tanani et al., 2023	Corrigendum to “Development and validation of Ran as a prognostic marker in stage I and stage II primary breast cancer”	Corrigendum; no independent cohort

S7. Included-study classification

Publication	Marker	Clinical source	Synthesis role
Yuen 2012	Direct RAN	Liverpool tissue + public data	Core quantitative/narrative
Yuen 2013	Direct RAN / MYC	Liverpool tissue + public data	Core stratification; overlapping Liverpool cohort
Yuen 2016	Direct RAN / MET	Liverpool tissue	Core stratification; overlapping Liverpool cohort
Sheng 2018	Direct RAN	Independent Chinese tissue cohort	Core quantitative
El-Tanani 2022	Direct RAN / MMP2	Untreated Liverpool subset	Core sensitivity/stratification
El-Tanani 2023	Direct RAN	Liverpool + public data	Core stratification; metric verification limitation
Vaidyanathan 2016	RAN pathway	Public datasets	Contextual nuclear-export target discovery
Behrens 2001	CSE1L/CAS	Human breast lesions	Contextual clinicopathology
Emberley 2002	RANBP9/RanBPM	Human invasive tumours	Contextual receptor/inflammation strata
Mehmood 2021	Transport factors	TCGA + microarrays	Contextual public-data prognosis
Siddig 2023	RANBP3L	ER-positive tissue cohorts	Contextual grade stratification
Dai 2023	XPOT	TNBC tissue + public data	Contextual target-specific prognosis
Hu 2024	RANBP3 methylation	Paired tissue/plasma	Contextual diagnosis
Hou 2025	XPOT	TCGA + METABRIC	Contextual prognosis/clinicopathology
Song 2025	RANBP1	TNBC tissue + public data	Contextual prognosis/immune strata

S8. Quantitative-synthesis rule after the updated search

No newly included pathway-marker report was added to the direct-RAN forest plots. Direct RAN, CSE1L, individual RAN-binding proteins and XPOT are distinct exposures and cannot be combined into one overall effect. The primary direct-protein display therefore remains one selected Liverpool estimate plus the

independent Sheng estimate. The GEO forest plots remain restricted to non-overlapping accession-level RAN mRNA effects for each endpoint. Public-data pathway studies were synthesized narratively and flagged for TCGA, METABRIC or GEO overlap.
